# Genetic Predisposition to MASLD: Potential for Therapeutic Management

**DOI:** 10.3390/ijms27041933

**Published:** 2026-02-18

**Authors:** Fani Karapanagiotidi, Chrysoula Boutari, Emmanouil Sinakos

**Affiliations:** 4th Department of Internal Medicine, Hippokration General Hospital, Aristotle University of Thessaloniki, 49 Konstantinoupoleos Street, 54642 Thessaloniki, Greece; fanikarap91@gmail.com (F.K.); em_sinakos@yahoo.com (E.S.)

**Keywords:** metabolic dysfunction-associated steatotic liver disease, PNPLA3, TM6SF2, MBOAT7, GCKR, HSD17B13

## Abstract

Metabolic Dysfunction-Associated Steatotic Liver Disease (MASLD) is now the most common liver disease worldwide, with a continuously increasing prevalence. The mechanisms involved in its pathophysiology are numerous and may include metabolic, environmental, and genetic factors. Genome-wide association studies have identified key genetic variants, most notably in PNPLA3, TM6SF2, MBOAT7, GCKR, and HSD17B13. This mini review discusses the mechanisms through which these variants contribute to the disease pathogenesis, an area that remains a rapidly evolving field of research. Beyond improving our understanding of MASLD, the identification of these variants may also aid in the development of targeted pharmacological approaches. We first summarize the major genetic variants associated with MASLD and then present findings from studies exploring how these variants may influence the efficacy of emerging pharmacotherapies. Finally, we examine the therapeutic agents in the field of precision medicine that are currently being tested in clinical trials. These therapeutic opportunities are a promising approach that may provide individualized solutions for this chronic liver disorder that affects a wide range of the population.

## 1. Introduction

Metabolic Dysfunction-Associated Steatotic Liver Disease (MASLD), previously referred to as non-alcoholic fatty liver disease (NAFLD), was first described in 1980 by Jurgen Ludwig et al. [1] as “non-alcoholic steatohepatitis.” The term MASLD was introduced to more accurately reflect its underlying pathophysiology. Today, it represents the most prevalent liver disorder worldwide, with global prevalence rising from 25% in 2016 to 30% in 2024 [2], and incidence continuing to increase. MASLD encompasses a spectrum from simple steatosis to Metabolic Dysfunction-Associated Steatohepatitis (MASH) (historically NASH), which represents the progressive inflammatory form of the disease and may further progress to fibrosis, cirrhosis, and hepatocellular carcinoma [2].

It is well established that the pathophysiology and progression of MASLD are strongly influenced by components of the metabolic syndrome, including obesity, type 2 diabetes, hypertension, and dyslipidemia, as well as by social and environmental factors such as ethnicity, smoking, sex, and age [3,4]. In recent years, with the development and expansion of human genome-wide association studies (GWAS), the importance of genetic variants and their underlying pathophysiology has come to the forefront. GWAS have opened new possibilities for treatment. In the era of precision medicine, identifying patients with certain genetic variants could make it possible to offer more personalized and targeted therapies. In this review, we summarize recent advances in the genetics of MASLD, beginning with an overview of the key genetic variants associated with disease susceptibility and progression, along with the underlying pathophysiological mechanisms they influence. We then explore the therapeutic implications of these findings, discussing both the influence of genetic variation on responses to existing therapies and the potential for precision treatments based on genetic profiling.

## 2. Genetic Variants Associated with MASLD

**Figure 1:**
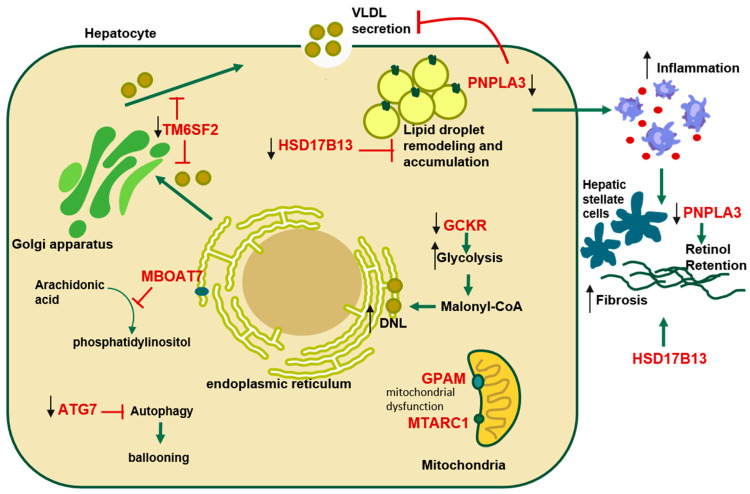
Key genetic variants and their pathophysiological role in the progression of the Metabolic dysfunction associated steatotic liver disease. Ιntracellular pathways through which key gene variants influence the pathophysiology of Metabolic Dysfunction-Associated Steatotic Liver Disease. PNPLA3 (patatin-like phospholipase domain–containing protein 3) and HSD17B13 (hydroxysteroid 17-beta dehydrogenase 13) impair lipid droplet remodeling, inflammation, and fibrogenesis; TM6SF2 (transmembrane 6 superfamily member 2) regulates very-low-density lipoprotein (VLDL) secretion; MBOAT7 (membrane-bound O-acyltransferase domain-containing 7) affects phosphatidylinositol production; GCKR (glucokinase regulatory protein) influences glycolysis and de novo lipogenesis (DNL) through the production of malonyl–coenzyme A (malonyl-CoA); MTARC1 (mitochondrial amidoxime reducing component 1) and GPAM (glycerol-3-phosphate acyltransferase, mitochondrial) impair mitochondrial function. ATG7 (autophagy-related 7) is associated with autophagy and hepatocellular ballooning, contribution to the progression of the disease.

Since the first GWAS related to MASLD was published in 2008 [5], several genetic variants have been identified and associated with the development and progression of the disease. Alongside other predisposing factors, these genetic susceptibilities contribute significantly to MASLD severity. The most important single nucleotide polymorphisms (SNPs) include Transmembrane 6 Superfamily Member 2 (TM6SF2), glucokinase regulatory protein (GCKR), membrane-bound O-acyltransferase domain-containing 7 (MBOAT7), hydroxysteroid 17-beta dehydrogenase 13 (HSD17B13), and the most extensively studied Patatin-Like Phospholipase Domain-Containing Protein 3 (PNPLA3) [3]. These genes have been associated with hepatic fat accumulation, fibrotic progression, and disturbances in lipid metabolism, playing a central role in both the onset and prognosis of MASLD (Figure 1).

### 2.1. PNPLA3

PNPLA3, located on chromosome 22, encodes a 481-amino acid protein with triglyceride hydrolase activity that is mainly expressed in adipose tissue, retina, and liver [6]. In 2008, Romeo et al. conducted the first GWAS in the context of MASLD and identified a strong association between the rs738409 C>G variant in PNPLA3, a substitution of isoleucine to methionine at position 148, and the hepatic fat content [5].

The I148M variant of PNPLA3 promotes hepatic steatosis through a complex gain-of-function mechanism rather than a simple loss of catalytic activity. This concept is supported by experimental models, where Pnpla3 knockout mice do not develop steatosis, whereas transgenic mice overexpressing the human I148M variant do [7]. This effect is partly explained by impaired ubiquitination and proteasomal degradation of the mutant protein, resulting in its accumulation on lipid droplets [8]. Although increased de novo lipogenesis is proposed as one of the mechanisms resulting in MASLD [9,10], some evidence suggests that this is not the case in PNPLA3 I148M carriers. A clinical and translational study observed that PNPLA3 I148M carriers exhibit reduced de novo lipogenesis, increased β-oxidation and ketogenesis, and a higher hepatic mitochondrial redox state compared with non-carriers, suggesting a distinct metabolic profile [11]. In addition, PNPLA3 has been linked to altered lipid export, as I148M carriers show reduced Very Low-Density Lipoprotein 1 (VLDL1) secretion compared with wild-type individuals for the same hepatic fat content, supporting a dual role of PNPLA3 in both lipid storage and export [12].

Beyond lipid metabolism, PNPLA3 has also been implicated in fibrosis progression. Pingitore et al. reported that livers from PNPLA3 I148M carriers show increased intracellular retinyl ester content in hepatic stellate cells compared with wild-type carriers. Together with impaired TGF-β mediated induction of PNPLA3 under liver injury, this mechanism may reduce retinol release and limit antifibrotic signaling [6].

Collectively, the I148M variant is associated with steatosis severity and disease progression across the MASLD spectrum, from simple steatosis to inflammation, fibrosis, and hepatocellular carcinoma [13,14]. The frequency of the PNPLA3 I148M allele varies significantly between ethnic groups, being highest among Hispanics, intermediate in European Americans, and lowest in African Americans. This distribution mirrors the observed prevalence of MASLD in these populations, supporting the role of genetic predisposition in disease susceptibility [7,15].

### 2.2. HSD17B13

HSD17B13 is a hepatic lipid droplet-associated protein involved in steroid hormone signaling and lipid droplet dynamics [15]. Several variants in or near HSD17B13 gene cause improper splicing, insertion, deletion, or nonsynonymous mutations and led to loss-of-function mutations [16].

HSD17B13 has been found to be highly expressed in MASLD patients [17]. In cultured hepatocyte cell lines, overexpression of HSD17B13 results in an increase in both the number and size of lipid droplets, possibly by stabilizing intracellular triglyceride content, whereas its suppression attenuated formation of lipid droplets [18]. Interestingly, unlike other risk variants, loss-of-function alleles in HSD17B13 are protective against MASLD. The protective role seems to be associated with retinol metabolism, via retinol dehydrogenase activity, inflammation and fibrogenesis rather than simply lipid accumulation in the liver. The rs72613567 variant was the first associated with reduced levels of alanine aminotransferase (ALT) and aspartate transaminase (AST), and further studies linked HSD17B13 loss-of-function alleles with lower risk of MASH, fibrosis, cirrhosis, and hepatocellular carcinoma (HCC) in MASLD patients [16].

### 2.3. TM6SF2

The impact of the TM6SF2 rs58542926 T allele was first highlighted in 2014 [19]. This missense variant replaces glutamate with lysine at residue 167 (E167K), resulting in a loss of function due to reduced protein levels in hepatocytes, despite comparable mRNA expression. The 167Lys variant is misfolded and undergoes accelerated intracellular degradation. TM6SF2 encodes a polytopic membrane protein localized mainly in the endoplasmic reticulum and Golgi apparatus, with catalytic activity as a sterol isomerase regulating hepatic VLDL secretion [3,15,19].

Loss of TM6SF2 function leads to reduced secretion of VLDL, triglycerides, and apolipoprotein B, resulting in hepatocellular lipid accumulation. Consequently, carriers of this variant tend to have increased liver fat content, and higher risk for MASH, advanced fibrosis, cirrhosis, and even HCC [15]. The variant has also been associated with elevated serum ALT levels, reflecting liver injury [19]. Nevertheless, the reduced circulating atherogenic lipoprotein burden in carriers contributes to decreased atherosclerosis and a lower risk of cardiovascular events. This highlights the allele’s dual role, causing liver damage and hepatic disease progression while potentially mitigating cardiovascular risk [20].

### 2.4. MBOAT7

The MBOAT7 rs641738 C>T variant was first identified as a genetic risk factor for alcoholic cirrhosis [21]. The rs641738 T allele is associated with reduced hepatic expression of MBOAT7, resulting in impaired phosphatidylinositol remodeling, increased free arachidonic acid, and enhanced hepatic inflammation. MBOAT7 encodes lysophosphatidylinositol-acyltransferase 1 (LPIAT1), a membrane protein mainly localized to the endoplasmic reticulum, lipid droplets, and mitochondria-associated membranes. This enzyme incorporates arachidonic acid (AA) and other unsaturated fatty acids into lysophosphatidylinositol, generating phosphatidylinositol. By channeling free AA into membrane phospholipids, MBOAT7 reduces the pool of free AA, which is available for pro-inflammatory eicosanoid synthesis, and thereby, negatively regulates Toll-like receptor signaling. Mechanistic studies showed that restoring MBOAT7 restoration in hepatocytes of MASH mice reduced levels of hepatocyte-TAZ (WWTR1), which is associated with the progression of fibrosis in MASH. Conversely, hepatocyte-MBOAT7 silencing enhanced TAZ upregulation in MASH [22]. A recent study found that changes in hepatocyte phospholipids due to MBOAT7 loss of function promote a cholesterol trafficking pathway that upregulates TAZ and the TAZ-induced profibrotic factor Indian hedgehog (IHH) [23].

Subsequent studies have demonstrated its impact across the entire spectrum of MASLD, including steatosis, inflammation, fibrosis, and even progression to hepatocellular carcinoma [15,21]. Nevertheless, the allele’s effect size is small compared to other genetic variants [15].

### 2.5. GCKR

The GCKR gene encodes an allosteric inhibitor of glucokinase (GCK), a key regulator of hepatic glucose metabolism [3]. The P446L variant (rs1260326 C>T SNP), leads to loss of GCKR function, which causes a reduction in the ability of GCKR in response to fructose-6-phosphate. This results in increased GCK activity without proper regulation, overstimulation of glycolysis and excessive production of malonyl-CoA [24]. This variant also reduces the sensitivity of the GCK-regulatory protein complex, resulting in increased activation of de novo lipogenesis, higher liver triglyceride content, and metabolic dysfunction. Thus, the P446L variant is related to steatosis, risk of MASLD and HCC, also observed in obese children and adolescents [15]. A different variant (rs780094) is linked to high triglyceride levels, development of MASLD, and more severe liver fibrosis [3].

### 2.6. Mitochondrial Amidoxime Reducing Component 1 (MTARC1)

MTARC1 encodes a mitochondrial enzyme located in the outer mitochondrial membrane and plays a role in detoxification. The rs2642438 variant has been identified to be protective against MASLD and cirrhosis, since it has been associated with improved mitochondrial function, reduced production of reactive oxygen species (ROS), and attenuation of lipid peroxidation in hepatocytes. MTARC1 encodes an enzyme involved in mitochondrial redox reactions; its protective allele appears to reduce oxidative stress and improve metabolic flexibility, contributing to a less fibrogenic hepatic environment [25] Given its mitochondrial localization, mARC1 may influence lipid trafficking via interactions with proteins such as TM6SF2 [26]. Moreover, rs2642438 is associated with reduced hepatic fibrogenesis and inflammatory activity [13] as mARC1 protein was observed in human hepatic CD68+ (macrophage) cells [26].

Except for the already mentioned variants and genes, several other mutations have been linked by GWAS to the progression of MASLD through distinct yet interrelated mechanisms. A missense variant (rs2792751, p.I43V) in the mitochondrial glycerol-3-phosphate acyltransferase (GPAM) gene, which encodes a key enzyme in triglyceride biosynthesis and is localized in the outer mitochondrial membrane, has been linked to increased liver fat and elevated transaminases, highlighting a role for mitochondrial dysfunction in MASLD pathogenesis [27]. Moreover, missense variants of mitochondrial DNA polymerase-γ (POLG) [28] and mitochondrially encoded cytochrome B (MT-CYB) [29], which are both linked to oxidative phosphorylation chain, are related with severe MASLD and MASH. Additionally, other genetic variants may influence MASLD progression through mechanisms such as inflammation, immune response, and transcriptional regulation. For example, a missense variant in PYGO1 has been associated with altered transcriptional pathways that could modulate hepatic inflammation and steatosis [30]. Finally, missense variants in *ATG7* (rs143545741, rs36117895), which is mainly localized in periportal hepatocytes, have been associated with autophagy impairment. These variants promote hepatocyte ballooning and inflammation, contributing thereby to the progression of MASLD [31]. Interestingly, a study of 832 obese children and adolescents identified another autophagy-related gene, the immunity-related GTPase family M (IRGM), and suggested that it contributes to MASLD development by altering hepatic lipid metabolism through the autophagy pathway [32]. Having discussed the genetic variants that influence MASLD pathogenesis, it is important to consider how these and other mutations may affect patient responses to current and emerging therapies.

## 3. Clinical Implications

### 3.1. Prediction of Therapeutic Response

Until the recent approval of the Thyroid hormone receptor beta (THRβ) agonist, Resmetirom [33], and the glucagon-like peptide-1 (GLP-1) receptor agonist, Semaglutide [34], treatment of MASLD was limited to lifestyle modification and weight loss strategies [35]. Shen’s et al. meta-analysis suggests that PNPLA3 I148M may modify the response to lifestyle interventions in MASLD. Some studies indicate that carriers of this allele, especially homozygotes, achieve greater reductions in hepatic steatosis with calorie restriction or exercise, although findings are inconsistent. Additionally, according to the same meta-analysis, as far as omega-3 supplementation is considered, the 148M variant has been linked to impaired polyunsaturated fatty acid mobilization, and while short-term trials showed no genotype-specific effect, the longer ones (>15 months) suggest that PNPLA3 I148M may influence treatment response. Overall, evidence supports a potential interaction between PNPLA3 genotype and dietary/lifestyle strategies, but current data remain limited and heterogeneous [36].

The pharmacological treatment landscape for MASLD is rapidly changing. New pharmacological agents, including fibroblast growth factor (FGF) 21 analogues, show promising results but are still under investigation in the context of MASLD/MASH treatment. Increasing evidence suggests, however, that genetic variants, and especially PNPLA3 I148M, may significantly influence therapeutic response, underscoring the importance of integrating genetics into treatment strategies.

#### 3.1.1. THRβ Agonists

THRβ is highly expressed in hepatocytes and is responsible for regulating lipid levels and metabolic pathways in the liver [37]. In March 2024, resmetirom, a THRβ selective agonist, received accelerated approval from the FDA for the treatment of MASH in patients with moderate to advanced liver fibrosis (F2–F3), based on the findings of the phase III MAESTRO-NASH clinical trial [38]. As part of the trial, an analysis was conducted in which single-nucleotide polymorphisms were genotyped in 740 patients to evaluate baseline characteristics and the response to resmetirom. Interestingly, a substantial proportion of patients carried genetic biomarkers, mainly PNPLA3, HSD17B13, and TM6SF2. While these variants were associated with differences in several baseline features, such as biochemical parameters, they were not associated with differential therapeutic response, whether assessed by liver biopsy, imaging, or other response markers [39].

#### 3.1.2. GLP-1 Agonists

GLP-1 receptor agonists, already established in Type 2 diabetes mellitus (T2DM) and obesity management, are under intensive investigation in the field of MASLD/MASH treatment. Semaglutide has shown promising results in a phase III trial, which included patients with biopsy-defined MASH and moderate/severe fibrosis, with both significant disease resolution and fibrosis improvement [40]. In a retrospective study of 220 patients with T2DM receiving semaglutide (subcutaneous or oral), genotyped for the PNPLA3 rs738409 variant, reductions in ALT levels after one year of treatment were more pronounced in G-allele carriers in both univariable and multivariable analyses. Effects on BMI and in non-invasive fibrosis indices (FIB-4) did not differ by genotype. The ALT reduction remained significantly greater in G-allele carriers even after 2 years of treatment [41].

Tirzepatide, in a phase II trial (SYNERGY-NASH) involving participants with MASH and moderate or severe fibrosis treated for 52 weeks, achieved higher rates of MASH resolution and fibrosis improvement compared with placebo [42]. A post hoc analysis of 131 patients showed that MASH resolution was more frequent among wild-type PNPLA3 carriers, whereas improvements in fibrosis, hepatic fat content, and body weight did not differ significantly by genotype. These findings suggest that the PNPLA3 G-risk allele may influence MASH development independently of hepatic steatosis [43].

Evidence on exenatide come from a small trial which suggested reductions in hepatic fat and ALT levels only in patients with the homozygous PNPLA3 148I genotype, whereas no such effects were seen in 148M carriers. Metabolic improvements occurred regardless of genotype [44]. Together, these findings highlight both the therapeutic potential of GLP-1 agonists and the modifying role of PNPLA3 variants on treatment response.

#### 3.1.3. FGF21 Analogues

FGF21 is an endocrine mediator that activates the co-receptor complex of β-klotho and FGF receptors, thereby regulating systemic energy homeostasis, lipid metabolism, and improving insulin sensitivity [45] Efruxifermin, a long-acting Fc–FGF21 analogue, is under phase III evaluation in the SYNCHRONY program. In the phase IIb BALANCED trial, efruxifermin given subcutaneously (28 mg or 50 mg) once per week improved fibrosis and resolved MASH in F2–F3 patients, alongside showing reductions in liver fat, triglycerides, and Pro-C3, compared to placebo [46]. Interestingly, a post-hoc analysis indicated efficacy across PNPLA3 genotypes with particularly pronounced histologic benefits in 148M homozygotes, while metabolic improvements (reductions in liver fat, serum triglycerides, and Pro-C3) were observed across genotypes, with greater improvements in insulin sensitivity and glycemic control among wild-type and heterozygous carriers Overall, efruxifermin appears effective in both genetically and metabolically driven MASH [47].

#### 3.1.4. Thiazolidinediones

Studies in patients with prediabetes or T2DM have shown that thiazolidinediones improve liver histology, reduce insulin resistance, and ameliorate fibrosis [2]. Lanifibranor, a pan-PPAR (peroxisome proliferator-activated receptor) agonist currently in phase III evaluation (NCT04849728), demonstrated dose-dependent improvements in MASH in the phase IIb NATIVE trial, alongside benefits in fibrosis, lipid profile, and glycemic control [48]. In this study, 247 non-cirrhotic MASH patients were randomized to lanifibranor or placebo for 24 weeks, of whom 219 were genotyped for PNPLA3 rs738409. Treatment response, including histologic and metabolic-immune markers, did not differ significantly by genotype, despite 148M carriers having more severe baseline disease. These results suggest that lanifibranor exerts beneficial effects across PNPLA3 genotypes [49].

### 3.2. Precision Therapeutic Interventions Based on Genetic Findings

In MASLD, advances in genomics have opened the door to therapies that target specific disease mechanisms, offering the potential for more effective and individualized care. These developments bring precision medicine closer to clinical practice, where treatment can be guided not only by histology and metabolic profile but also by the patient’s genetic background. RNA interference (RNAi) is a promising therapeutic avenue for many diseases. It can be approached by antisense oligonucleotides (ASOs), small interfering RNA (siRNA), or short hairpin RNA [7]. Since the gain-of-function mutation in PNPLA3 is a key driver for MASLD, therapies that specifically target the mutant PNPLA3 I148M protein could represent a promising therapeutic approach. In this context, in 2019, Linden et al. tested ASO-mediated silencing of Pnpla3 in homozygous Pnpla3 148M mice. In mice fed a MASH-inducing diet, this treatment significantly reduced inflammation and fibrosis, suggesting that this approach may prove beneficial in terms of all aspects of MASLD [50]. Nowadays, based on variants identified in the past years by GWAS, there are therapeutic approaches investigated in clinical trials (Table 1).

#### 3.2.1. PNPLA3 Targeted Therapies

AZD2693, a GalNAc-conjugated ASO designed to selectively silence hepatic PNPLA3 expression, was first evaluated in preclinical models, including human hepatocytes and mice expressing human PNPLA3, resulting in reduction in the expression of PNPLA3. Subsequently, two phase I studies were conducted comprising ascending doses in humans, demonstrating an acceptable safety and tolerability profile. They achieved suppression of PNPLA3 expression, with knockdown approaching 90% at higher doses, with changes in hepatic steatosis, a dose-dependent rise in polyunsaturated fatty acids in serum triglycerides and reductions in inflammatory markers [51]. A phase IIb trial in patients with F0–F3 fibrosis has been completed; however, no results have been published to date (NCT05809934).

ARO-PNPLA3 (JNJ-75220795), another GalNAc-siRNA evaluated in two phase 1 trials including single and ascending doses, respectively [52]. Notably, higher doses achieved up to 40% liver fat reduction at 12 weeks in homozygous carriers, with effects sustained over 24 weeks. Effects were not observed in heterozygous carriers. The treatment was generally well tolerated, with no clinically meaningful safety concerns [52]. LY3849891 is another PNPLA3 GalNac-siRNA, currently undergoing a phase I trial with single and repeated subcutaneous ascending doses, assessing its safety and tolerability in individuals with MASLD (NCT05395481). Additional early-stage candidates include ALN-PNP and the oral small-molecule degrader PF-07853578 (NCT05890105), both in phase I development.

#### 3.2.2. HSD17B13 Targeted Therapies

The protective effect of certain loss-of-function variants in HSD17B13 against MASLD has highlighted this enzyme as a promising therapeutic target. Consequently, pharmacologic inhibition of HSD17β13 is being explored as a potential strategy, and several therapeutic agents are currently tested in clinical trials [53]. ARO-HSD is a GalNAc-conjugated siRNA designed to selectively reduce hepatic HSD17B13 mRNA expression in hepatocytes. In a phase Ι clinical study including healthy volunteers and patients with confirmed or suspected ΜASH, it was generally well tolerated, without serious adverse events. Short-term administration resulted in a mean reduction of hepatic HSD17β13 mRNA and protein levels of approximately 75%, accompanied by significant decreases in ALT (NCT04202354) [54]. Based on these results, its effect on histology is now being tested in the phase IIb HORIZON trial in individuals with MASH and biopsy-confirmed F3-F4 fibrosis (NCT05583344).

Rapirosiran (ALN-HSD), another GalNAc-siRNA targeting HSD17B13, was evaluated in a phase I clinical study. In first part, which included healthy volunteers, no serious adverse effects were reported. Its second part included two doses administrated in individuals with MASH, resulting in a dose-dependent reduction in hepatic HSD17B13 mRNA, reaching at the same time a median decrease of 78% at 6 months in the highest-dose group [55]. Building on these findings, the ongoing phase II trial (NCT05519475) is now investigating rapirosiran in patients with MASH and F2–F3 fibrosis to assess its impact on histological resolution of steatohepatitis and fibrosis progression.

#### 3.2.3. MTARC1 Targeted Therapies

Finally, other agents such as NN6581, a GalNAc-siRNA against MTARC1 (NCT05599945), has been evaluated in healthy participants and participants with hepatic steatosis in a phase I trial, with results awaited.

All these advances highlight a shift toward genotype-driven therapies that may not only slow or inhibit disease progression but also offer a more personalized treatment approach in the field of MASLD.

## 4. Future Research Directions

Although noticeable progress in defining genetic variants associated with MASLD has been achieved, there are still important gaps regarding their functional interpretation and therapeutic applications. Thus, future research should focus on integrative multi-omics approaches involving genomics, epigenomics, transcriptomics, proteomics, and metabolomics to clarify how risk alleles such as PNPLA3, TM6SF2, MBOAT7, and HSD17B13 are related to metabolic and inflammatory pathways across each disease stage. Single-cell and spatial transcriptomic technologies could further clarify cell-type-specific effects of these variants within hepatocytes, hepatic stellate cells, and immune populations, thereby contributing to better mechanistic understanding of fibrosis progression and resolution, as well [56].

Another future target could be the development of genotype-based therapeutic strategies and precision medicine systems. Pharmacogenomic studies are required to evaluate whether genetic risk profiles are able to predict response to emerging treatments, such as PPAR modulators and RNA-based therapies. Particularly, antisense oligonucleotides or small interfering RNA targeting PNPLA3 expression have shown promising results in preclinical and early clinical studies, emphasizing the feasibility of directly modulating genetically driven disease pathways [57]. Finally, future clinical trials performing genetic stratification may optimize patient selection, therapeutic efficacy, and safety, assuring personalized management of MASLD.

## 5. Conclusions

Recent advances in GWAS have greatly improved our understanding of the genetic basis of MASLD, leading to the discovery of key genes and variants that influence disease risk and progression. MASLD is a complex condition influenced by metabolic syndrome, environmental factors, and biological pathways such as lipid droplet metabolism, VLDL secretion, and hepatic lipogenesis. Understanding these mechanisms is now helping to bridge the gap between basic science and clinical practice, paving the way for targeted and genotype-based treatments. Precision medicine approaches, together with efforts to match existing therapies to patient genetic profiles, have the potential not only to improve treatment responses but also to prevent disease in those at highest risk. In order to accomplish this demanding target, functional validation of the existing evidence of the impact of genetic variation on MASLD treatments is warranted. If this is achieved through late-phase clinical trials, incorporating genetic screening into routine care and clarifying which patients benefit most from each intervention could ultimately mark the start of a new era in MASLD management, finally providing effective and specific solutions for a disease that has long lacked them.

## Figures and Tables

**Table 1:** Investigational precision medicine therapeutics in MASH, classified by molecular target, therapeutic category, and clinical development phase.

Drug	Category	Target	Clinical Phase	Participants (n)	Primary Endpoints	Geographic Region	Clinical Trial ID
PNPLA3 Targeted Therapies							
AZD2693	GalNAc-ASO (Antisense Oligonucleotide)	PNPLA3 mRNA knockdown	IIb	220	MASH resolution without fibrosis worsening	International	NCT05809934 (Completed)
ARO-PNPLA3 (JNJ-75220795)	GalNAc-siRNA	PNPLA3 mRNA silencing	I	55	Safety and tolerability	USA	NCT05039710 (terminated), NCT04844450 (completed)
LY3849891	GalNAc-siRNA	PNPLA3 mRNA silencing	I	≈176	Safety and MRI-based PD endpoints	International	NCT05395481 (active, not recruiting)
ALN-PNP	GalNAc-siRNA	PNPLA3 mRNA silencing	I	≈156	Safety and tolerability	USA	NCT05648214 (recruiting)
PF-07853578	Small-Molecule Targeted Degrader	PNPLA3 protein degradation	I	23	Safety and tolerability	USA	NCT05890105 (completed)
AMG 609	GalNAc-siRNA	PNPLA3 mRNA silencing	I	47	Safety and tolerability	International	NCT04857606 (completed, Discontinued)
HSD17B13 Targeted Therapies							
ARO-HSD	GalNAc-siRNA	HSD17B13 mRNA silencing	II	284	Histological fibrosis improvement and MASH resolution	International	NCT05583344 (Active, not recruiting)
Rapirosiran (ALN-HSD)	GalNAc-siRNA	HSD17B13 mRNA silencing	II	≈90	Change from baseline in qFibrosis score (SHG/TPE microscopy)	International	NCT05519475 (Recruiting)
AZD7503	GalNAc-ASO	HSD17B13 mRNA knockdown	I	56	Safety and tolerability	USA	NCT05143905 (completed)
MTARC1 Targeted Therapies							
NN6581	GalNAc-siRNA	MTARC1 mRNA silencing	I	48	Safety and tolerability	UK	NCT05599945 (Active, not recruiting)

Abbreviations: ASO: antisense oligonucleotide; GalNAc: N-acetylgalactosamine conjugate; HSD17B13: hydroxysteroid 17-beta dehydrogenase 13; MTARC1: Mitochondrial amidoxime reducing component 1; PD: Pharmacodynamics; PNPLA3: patatin-like phospho-lipase domain-containing protein 3; qFibrosis: quantitative liver fibrosis; SHG: second harmonic generation; siRNA: small interfering RNA; TPE: two-photon excitation.

## Data Availability

No new data were created or analyzed in this study. Data sharing is not applicable.
